# 4-*O*-Carboxymethylascochlorin Inhibits Expression Levels of on Inflammation-Related Cytokines and Matrix Metalloproteinase-9 Through NF–κB/MAPK/TLR4 Signaling Pathway in LPS-Activated RAW264.7 Cells

**DOI:** 10.3389/fphar.2019.00304

**Published:** 2019-03-27

**Authors:** Junyoung Park, Sun-Hyung Ha, Fukushi Abekura, Hakseong Lim, Juni Magae, Ki-Tae Ha, Tae-Wook Chung, Young-Chae Chang, Young-Choon Lee, Eunyong Chung, Jiyeon Ku, Cheorl-Ho Kim

**Affiliations:** ^1^Molecular and Cellular Glycobiology Unit, Department of Biological Sciences, Sungkyunkwan University, Suwon, South Korea; ^2^Magae Bioscience Institute, Tsukuba, Japan; ^3^Division of Applied Medicine, School of Korean Medicine, Pusan National University, Yangsan, South Korea; ^4^School of Korean Medicine and Healthy Aging Korean Medical Research Center, Pusan National University, Yangsan, South Korea; ^5^Research Institute of Biomedical Engineering, Department of Medicine, School of Medicine, Catholic University of Daegu, Daegu, South Korea; ^6^Department of Medicinal Biotechnology, College of Health Science, Dong-A University, Busan, South Korea; ^7^Department of Anesthesiology and Pain Medicine, Bucheon St. Mary’s Hospital, College of Medicine, The Catholic University of Korea, Seoul, South Korea

**Keywords:** AS-6, inflammation, MMP, TLR, LPS, RAW macrophage cells

## Abstract

Toll-like receptor 4 (TLR4) and matrix metalloproteinase-9 (MMP-9) are known to play important roles in inflammatory diseases such as arteriosclerosis and plaque instability. The purpose of this study was to perform the effect of 4-*O*-carboxymethylascochlorin (AS-6) on MMP-9 expression in lipopolysaccharide (LPS)-induced murine macrophages and signaling pathway involved in its anti-inflammatory effect. Effect of AS-6 on MAPK/NF-κB/TLR4 signaling pathway in LPS-activated murine macrophages was examined using ELISA, Western blotting, reverse transcription polymerase chain reaction (RT-PCR) and fluorescence immunoassay. MMP-9 enzyme activity was examined by gelatin zymography. AS-6 significantly suppressed MMP-9 and MAPK/NF-κB expression levels in LPS-stimulated murine macrophages. Expression levels of inducible nitric oxide synthase (iNOS), COX2, MMP-9, JNK, ERK, p38 phosphorylation, and NF-κB stimulated by LPS were also decreased by AS-6. Moreover, AS-6 suppressed TLR4 expression and dysregulated LPS-induced activators of transcription signaling pathway. The results of this study showed that AS-6 can inhibit LPS-stimulated inflammatory response by suppressing TLR4/MAPK/NF-κB signals, suggesting that AS-6 can be used to induce the stability of atherosclerotic plaque and prevent inflammatory diseases in an *in vitro* model.

## Introduction

Inflammation is the body’s defensive response to a stimulus (Klasing, 1991). While general inflammation leads to tissue protection and injury regeneration in response to injury and infection, chronic inflammation usually leads to loss of immune system, resulting in tissue damage and the occurrence of various diseases. Inflammation is linked to various chronic diseases such as vascular dysfunction, heart diseases, cancers, neurological disorders, obesity, diabetes, and atherosclerotic plaques (Jayson, 1989).

Matrix metallopeptidases play a crucial role in cell behaviors including host defense, differentiation, migration, angiogenesis, cell proliferation and apoptosis. MMP-9 can destroy the ECM. It is prominently marked in vulnerable areas of atherosclerotic plaque (Hernandez-Barrantes et al., 2002; Murphy et al., 2002). MMP-9 is prominently marked in the vulnerable areas of the atherosclerotic plaque (Kalela et al., 2000; Arihiro et al., 2001). Pro-inflammatory cytokine and MMP-9 are important factors that provide plaque the decomposition of ECM. MMP is also known to be the main cause of atherosclerosis.

Toll-like receptors are known to control initial inflammatory responses. They play a major role in inducing activation of the innate immune system (Cen et al., 2018). TLR4, a member of the TLRs, has been extensively studied in atherosclerosis. It is known to play an important role in plaque instability (Croce and Libby, 2007). LPS-activated TLR4 can induce MAPKs and NF-κB pathway. In addition, induction of TLR4 pathway is required for LPS-stimulated expression of inflammatory factors including MMP, COX-2 and iNOS (Billack, 2006).

**Figure 1:**
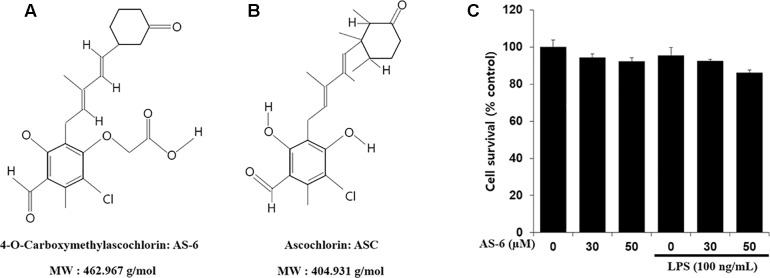
Chemical structures of AS-6 and ascochlorin (ASC) and effects of AS-6 on viability of RAW 264.7 cells. **(A)** 4-*O*-Carboxymethylascochlorin: AS-6 (Chemical structure). **(B)** Ascochlorin: ASC (Chemical structure). **(C)** Cell viability measured by MTT assay. 1 × 10^4^ cell/well cells were treated with 0, 30, or 50 μM of AS-6 and LPS (100 ng/ml) for 24 h.

Phenyl-phenolic compounds are known to have antiviral, anticancer, and antimicrobial activities as physiologically active substances. Ascochlorin (ASC, Figure 1B) has been isolated from fungus Ascochyta viciae. It has been reported that ASC and its derivative compounds possess strong antifungal and antioxidative activities (Tamura et al., 1968; Togashi et al., 2003). In addition, they can suppress transforming growth factor β1-activated plasminogen activator inhibitor -1 expression by inhibiting phospho- epidermal growth factor receptor in rat kidney fibroblast cells (Cho et al., 2012). Recently studies have studied that methylated derivative of ascochlorin (AS-6, Figure 1A) can cause ER stress and activate autophagy in human hepatocellular carcinoma cells (Kang et al., 2012). It has also been reported that AS-6 can inhibit TNF-α-stimulated chemokine and adhesion molecule expression in vascular smooth muscle cell lines and protect against microglial-associated neurotoxicity in BV2 and SH-SY5Y co-cultured cells (Park et al., 2006, 2018). We have previously reported that structurally derivative compounds ascochlorin (ASC) and ascofuranone (AF) exhibit anti-inflammatory effects (Lee et al., 2016; Park et al., 2017). During our serial studies of ASC compounds, it has been found that AS-6 can stabilize HIF-1α though AMPK induction and activate apoptosis in comparison with apoptotic activators in human leukemia cells (Jeong et al., 2011). However, it is currently unknown whether AS-6 can regulate inflammatory reaction of macrophages. How AS-6 mediates the inflammatory reaction at the basic cellular membrane level is unclear either. Therefore, the purpose of this study was to examine the effect of AS-6 on MMP-9 expression in LPS-stimulated murine macrophages and signaling included in its effect of anti-inflammation.

In this study, we found that AS-6 could inhibit behavioral responses linked to inflammatory mediators. AS-6 also suppressed intracellular signaling, especially gene expression of inflammatory cytokines. Studies of its molecular mechanisms showed AS-6’s anti-inflammatory effect.

## Materials and Methods

### Cell Culture and MTT Assay

RAW 264.7 macrophage cell line was purchased from the American Type Culture Collection (ATCC, Rockville, MD, United States). These cells were cultured in (DMEM, Rockville, MD, United States) supplemented with 10% FBS and 1% antibiotic-antimycotic solution (Gibco, United States). Cells were grown in a humidified atmosphere containing 5% CO_2_ at 37°C. To assess cell viability, RAW 264.7 cells were seeded into a 96-well plate at a density of 1 X 10^4^ cells/well and treated with 0, 30, or 50 μM of AS-6. MTT solution was added and incubated for 4 h at 37°C in a CO_2_ incubator. The reaction product was solubilized with dimethyl sulfoxide (DMSO) and optical density was measured at 550 nm after 15 min of incubation.

### Reagents

4-*O*-carboxymethylascochlorin was isolated from a fungal strain of *Ascochyta viciae* provided by Dr. Young-Chae Chang, Daegu, South Korea and Dr. Junji Magae, Tokyo, Japan. The phytopathogenic fungus Ascochyta viciae LIBERT was obtained from the agricultural department and deposited at Mage Bioinstitute, Tokyo, Japan. AS-6 was dissolved in DMSO as a 50 mmol/L stock solution and diluted to appropriate concentration with medium. The final concentration of DMSO was adjusted to 0.1% (v/v) in the culture media. Griess reagent, Hoechst staining solution, LPS (*Escherichia coli* 0111:B4), and MTT were purchased from Sigma-Aldrich (St. Louis, MO, United States). Specific antibodies are listed in Table 1.

**Table 1:** Antibody sources and concentrations.

Antigen	Cat. no	Dilution	Manufacturer
iNOS	sc-651	1:1000	SantaCruz
			Biotechnology
β-actin	sc-47778	1:2000	SantaCruz
			Biotechnology
COX-2	sc-1745	1:2000	SantaCruz
			Biotechnology
p38	sc-535	1:1000	SantaCruz
			Biotechnology
p-p38	sc-7975	1:1000	SantaCruz
			Biotechnology
TLR4	sc-293072	1:1000	SantaCruz
			Biotechnology
NF-κB	sc-372	1:2000	SantaCruz
			Biotechnology
p-ERK	9101S	1:1000	Cell Signaling
			Technology
ERK	9101S	1:1000	Cell Signaling
			Technology
p-JNK	9255S	1:2000	Cell Signaling
			Technology
JNK	9252S	1:2000	Cell Signaling
			Technology
MyD-88	4615	1:1000	Cell Signaling
			Technology

### Nitric Oxide (NO) Assay

RAW 264.7 cells were seeded into 24-well plates at density of 1 × 10^5^ cells/well. Cells were treated with 100 ng/mL LPS alone or with various doses of AS-6 for 24 h. After cells were incubated in a CO_2_ incubator at 37°C for 24 h, NO production was determined by assessing nitrite level in culture media. This was executed by mixing the medium with Griess reagent. Optical density was then measured at 540 nm after 10 min of incubation.

### Measurement of Pro-inflammatory Cytokines and Prostaglandin E2

Levels of pro-inflammatory cytokines such as TNF-α, IL-6, and IL-1β in culture medium were assessed using ELISA kits (Affymetrix, eBioscience) with each cytokine-specific antibody following the manufacturer’s recommended protocol. PGE_2_ production in cell culture medium was assessed by ELISA assay kit (Cayman Chemical) following the manufacturer’s recommended protocol.

### Western Blot Analysis

RAW cells (5 × 10^5^ cells/well) were treated without or with AS-6 (0–50 μM) and LPS (100 ng/mL) for 24 h. For Nuclear protein separation and MAPKs, to assess whether AS-6 affected MAPK phosphorylation in LPS-treated murine macrophages, cells were pre-treated with AS-6 after LPS stimulation and then levels of p-p38, p-ERK and p-JNK were assessed. After incubation, cells were rinsed three times with PBS (pH 7.4). Whole-cell extracts were prepared using cell lysis buffer [50 mm Tris, 150 mm NaCl, 5 mm ethylenediaminetetraacetic acid (EDTA), 1 mm dithiothreitol (DTT), 0.5% NP-40, l M phenylmethylsulfonyl fluoride, l M aprotinin, and l M leupeptin, adjusted to (pH 8.0)] supplemented with protease inhibitor cocktail on ice for 15 min. Cell remains were scraped and centrifuged for 10 min. Cytosolic and nuclear fractions of proteins were isolated using NE-PERTM nuclear and cytoplasmic extraction reagents (Thermo Scientific) following the manufacturer’s manual. Protein concentration was assessed by Bio-Rad protein assay (Bio-Rad Laboratories, Hercules, CA, United States). Proteins (15–40 μg) were separated by 10–15% SDS-polyacrylamide gel and transferred to nitrocellulose (NC) membranes. Each membrane was blocked with 5% skim milk in Tris-buffered saline (150 mm NaCl, 10 mm Tris–Hcl, pH 7.5) containing 0.01% Tween-20 (TBS–T buffer). To observe target proteins, the membrane was incubated with primary antibodies specific for iNOS, COX-2, NF-κB, p-ERK1/2, ERK1/2, p-p38, p38, p-JNK, and JNK overnight at 4°C. The membrane was then rinsed with TBS–T buffer and incubated with horseradish peroxidase (HRP)-conjugated anti-mouse, anti-rabbit, or anti-goat immunoglobulin G secondary antibodies. Membrane was developed using ECL reagent. Images were analyzed using a western imaging system ChemiDOC (Davinch-K, Davinch-*In vivo*^TM^). Membranes can be re-used after 15 min of stripping in Reblot solution (25 mm glycine–Hcl, pH 2, 1% (w/v) SDS) at room temperature.

### Gelatin Zymography

Cultural medium with or without specific treatment was collected from 12-well dishes. The sample was mixed with a 5% Tris–glycine SDS sample buffer and loaded into 10% polyacrylamide gel with 1 mg/ml gelatin. After running at 110V for 80 min, the gel was washed with 2.5% Triton X-100 for 20 min and then incubated with 10 mm Tris Base, 40 mm Tris–HCl, 200 mm NaCl, 10 mm CaCl_2_ incubation buffer at room temperature for 12 h. The gel was then dyed for 2 h with 0.5% Coomassie blue in 50% methanol and 10% glacial acetic acid in 40%. MMP-9 activity area that appeared as a transparent band was detected from a blue background.

### Reverse Transcription-Polymerase Chain Reaction (RT-PCR) Analysis

Total RNAs were extracted from RAW cells using TRIzol reagent (Invitrogen) and converted to cDNA using an RT-PCR system (total RNA, 1 μg). Target gene amplification was executed using gene-specific oligonucleotide primers in a common PCR system. Specific RT-PCR primers are listed in Table 2. PCR products were assessed on 1.5% agarose gels and bands were visualized after ethidium bromide staining. The band density was quantified by scanning the gel with a gel documentation and analysis system (Image J, Bethesda, MD, United States).

**Table 2:** List of primers used for RT-PCR.

Gene	Sense primer (5′–3′)	Antisense primer (5′–3′)
iNOS	5′-ATGTCCGAAGCAAACATCAC-3′	5′-TAATGTCCAGGAAGTAGGTG-3′
COX-2	5′-GGAGAGACTATCAAGATAGT-3′	5′-ATGGTCAGTAGACTTTTACA-3′
MMP-9	5′-CCTGTGTGTTCCCGTTCATCT-3′	5′ -CGCTGGAATGATCTAAGCCCA-3′
TNF	5′-TCAGCCTCTTCTCATTCCTG-3′	5′-TGAAGAGAAC-CTGGGAGTAG-3′
IL-1β	5′-TGCAGAGTTCCCCAACTGGTACA-3′	5′-GTGCTGCCTAATGTCCCCTT-G-3′
IL-6	5′-CCGGAGAGGAGACTTCACAG-3′	5′-TCCACGATTTCCCAG-AGAAC-3′
β-actin	5′-GATCCGTGAAGATCAAGATCATTGCT-3′	5′-TGATCTTCATTTTTTACGCGTGAATT-3′

### Immunofluorescence Assay

RAW cells were cultured in 24-well pates at concentration for 12 mm-diameter sterile coverage, treated with AS-6 (50 μM) for 15 min, and incubated for another 30 min. Cells were fixed with 4% paraformaldehyde in PBS for 20 min, rinsed with PBS three times, and incubated with 0.2% Triton X-100 in PBS for 37°C. Non-specific coupling was blocked with 1% bovine serum albumin in PBS for 1 h at 37°C. Factor-specific antibody (1:500, 1% BSA in PBS) was then added and incubated overnight at 4°C to study in-cell transformation of NF-κB. After rinsing with 0.1% Tween-20 in PBS, cells were further incubated with a FITC-conjugated anti-rabbit IgG antibody for 3 h at 4°C. For nuclear staining, Hoechst solution was added at final dose of 0.5 μg/mL and incubated at room temperature for 10 min. After rinsing with PBS-T, the slide was equipped with an anti-fade reagent (molecular probe). Images were then analyzed using a fluorescence microscope (LSM 700, AxioObserver, C-Apochromat 63x/1.20 W Korr M27).

### Statistical Analysis

All experiment results are representative of at least three independent experiments performed in triplicate. The results of the data were analyzed by one-way analysis of variance (ANOVA), followed by following by *post hoc* Bonferroni test; *p*-values of <0.05 were considered statistically significant. ^∗^*p* < 0.05 and ^∗∗^*p* < 0.01 indicate significant differences from the LPS alone-treated cells.

## Results

### Effects of AS-6 on Cell Viability and Production of PGE_2_ and NO

**Figure 2:**
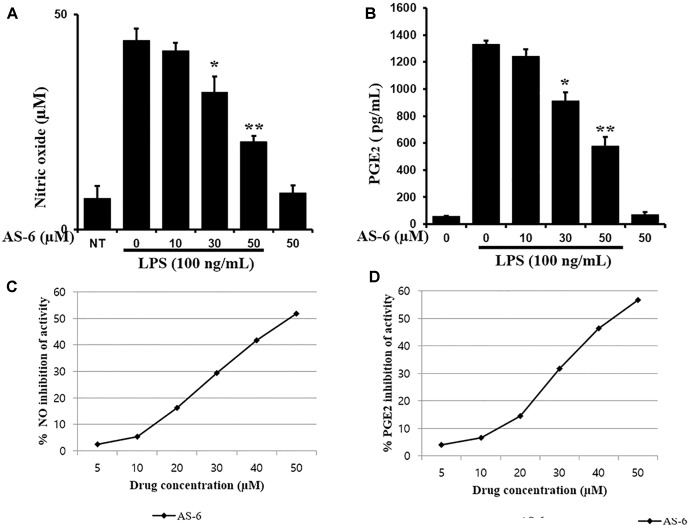
Effects of AS-6 on PGE2 and NO production in macrophage cells. **(A)** 5 × 10^5^ cells/well cells were treated with LPS (100 ng/ml) alone or with AS-6 (0–50 μM) for 24 h. NO in the medium was assessed using Griess assays. **(B)** 5 × 10^5^ cells/well cells were treated with LPS (100 ng/ml) alone or with AS-6 (0–50 μM) for 24 h. PGE_2_ in the medium was assessed by ELISA. **(C,D)** Inhibition curves of the AS-6-treated NO and PGE2 productions at the different concentrations (0, 5, 10, 20, 30, 40, and 50 μM). Results shown are representative of three independent experiments. They are presented as mean ± SEM. ^∗^*p* < 0.05 and ^∗∗^*p* < 0.01, significant differences from LPS treated cells. NT, no treatment.

The effect of AS-6 on viability of RAW macrophages in the presence or absence of LPS was assessed. AS-6 did not cause significant cytotoxicity at concentrations up to 50 μM in the presence or absence of 100 ng/mL LPS (Figure 1C). Therefore, its anti-inflammatory effects were tested at non-cytotoxic concentrations of 30 to 50 μM AS-6. Next, the effect of AS-6 were investigated by evaluating levels of NO and PGE_2_ production after LPS activation (Downey et al., 1988; Sadowska-Krowicka et al., 1998; Laroux et al., 2001; Guastadisegni et al., 2002). NO and PGE_2_ tests were examined as described in the Section “Materials and Methods”. The basic level of NO was produced in murine macrophage cells without LPS treatment from a 24-well plate at a dose of 1 × 10^5^ cells/well. Treating macrophages with AS-6 without LPS stimulation did not affect the production of NO or PGE2. However, AS-6 dose-dependently inhibited LPS-stimulated production of PGE_2_ and NO (Figure 2A,B) and the inhibitory activities of AS-6 were examined at different concentrations of 0, 5, 10, 20, 30, 40, and 50 μM AS-6 on NO and PGE2 productions (Figure 2C,D), respectively.

### Effect of AS-6 on Gene and Protein Expression Levels of MMP-9, COX-2, and iNOS

**Figure 3:**
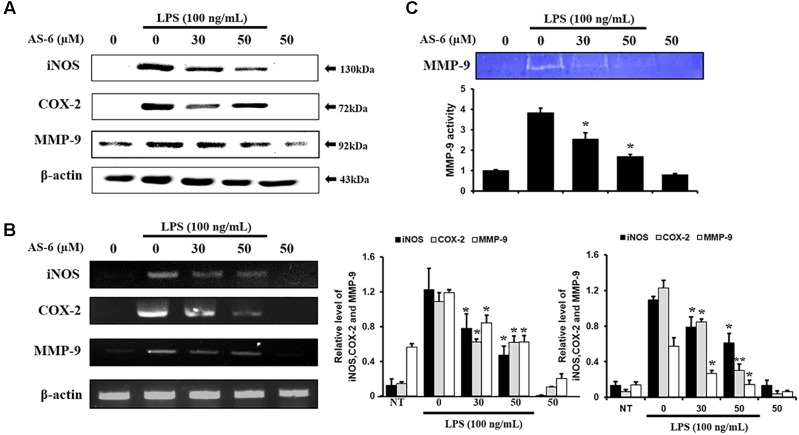
Effect of AS-6 on protein and expression levels of iNOS, COX-2, and MMP-9 in macrophage cells. RAW 264.7 cells were treated with 050 μM AS-6 and then co-treated with 100 ng/mL LPS for 24 h. **(A,B)** Protein and mRNA levels were examined by Western blot and RT-PCR, respectively. **(C)** Zymography results of MMP-9 activity. Results shown are representative of three independent experiments. They are presented as mean ± SEM. ^∗^*p* < 0.05 and ^∗∗^*p* < 0.01, significant differences from the LPS treated cells. NT, no treatment.

To evaluate anti-inflammatory effects of AS-6, protein expression levels of MMP-9, COX-2, and iNOS in murine macrophages stimulated by LPS were evaluated by RT-PCR and Western blotting. After 24 h of incubation of RAW cells with 100 ng/ml LPS and 0–50 μM AS-6, protein and mRNA expression levels were evaluated. As shown in Figure 3A, protein expression levels of MMP-9, iNOS and COX-2 in murine macrophages treated with AS-6 were decreased. In cells with AS-6 treatment at high concentrations in the range of 0 to 50 μM, COX-2, iNOS and MMP-9 mRNA levels were decreased (Figure 3B). In murine macrophage, LPS-induced MMP-9 was significantly lower than that in control cells as shown in Figure 3C. These results showed that mRNA and protein levels of MMP-9, COX-2 and iNOS and MMP-9 enzyme activity were decreased by treatment with AS-6.

### Effects of AS-6 on Levels of IL-6, TNF-α, and IL-1β Cytokines Activated by LPS

**Figure 4:**
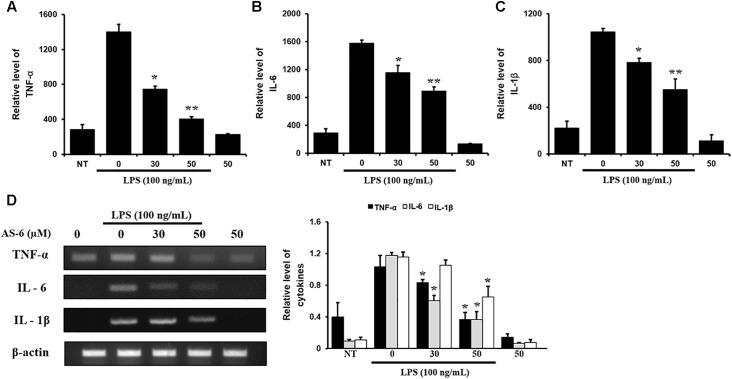
Effects of AS-6 on LPS-induced TNF-α, IL-6, and IL-1β cytokine levels in macrophage cells. Macrophage cells were treated with 0–50 μM AS-6 and then co-treated with 100 ng/mL LPS for 24 h. **(A–C)** 0–50 μM AS-6 dose-dependently attenuated LPS-induced transcription levels and TNF-α, IL-1β, and IL-6 inflammatory cytokines using ELISA. **(D)** mRNA levels of cytokines levels of TNF-α, IL-6, and IL-1β were analyzed by RT-PCR. Results shown are representative of three independent experiments. Results are presented was mean ± SEM. ^∗^*p* < 0.05 and ^∗∗^*p* < 0.01 indicate significant differences from LPS treated cells. NT, no treatment; L, LPS; L+A, LPS + AS-6; A, AS-6.

Effects of AS-6 on extracellular secretions of renowned proinflammatory cytokines such as IL-6, TNF-α and IL-1β (Miossec, 1991; Stein and Gordon, 1991), were determined by RT-PCR. Each cytokine specific antibody was used in ELISA. Treatment of AS-6 decreased LPS-activated transcription levels and inflammatory cytokines including IL-1β TNF-α and IL-6 in a dose-dependent manner (Figure 4A–D). These results showed that AS-6 suppressed the secretions and expression of significant pro-inflammatory cytokines including IL-1β, TNF-α, and IL-6 in murine macrophages.

### Effect of AS-6 on LPS-Stimulated Nuclear Translocation of NF-kB

**Figure 5:**
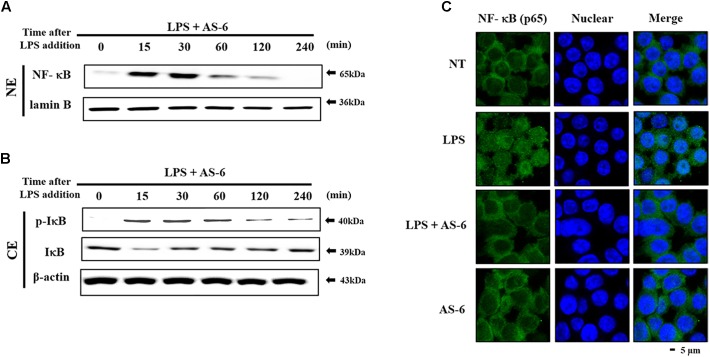
Effects of AS-6 on LPS-stimulated NF-kB nuclear translocation in RAW macrophage cells. **(A,B)** RAW cells were pre-treated with AS-6 (30 μM) for 60 min before treatment with 100 ng/mL LPS for 0–240 min time dependently. Cells were harvested and separated into nuclear and cytosolic extracts. Nuclear extracts were subjected to translocation to the nuclear region of NF-κB subunit by Western blot. **(C)** Transfer of NF-κB to the nucleus was detected by immunofluorescence assay. Macrophage cells were immune-stained by FITC and Hoechst. Scale bars, 5 μm. NE, Nuclear extracts; CE, Cytosolic extract; A, AS-6; L, LPS; L+A, LPS with AS-6; NT, no treatment.

Nuclear factor kappa-light-chain-enhancer of activated B cells is a crucial transcription factor associated with pro-inflammatory responses (Hietaranta et al., 2004; Ahn and Aggarwal, 2005; Viatour et al., 2005). We determined whether AS-6 could modulate LPS-treated NF-κB nuclear translocation in murine macrophages. AS-6 attenuated the nuclear translocation from the cytosol time-dependently (Figure 5A). IκB, an NF-κB regulator, was evaluated to determine the level of reduction of nuclear translocation. As expected, degradation of IκB expression and lowered the p-IκB expression level (Figure 5B). These results showed that AS-6 could inhibit LPS-stimulated the nuclear translocation in RAW 264.7 cells. To confirm the reduction in the translocation of NF-κB, we measured intercellular translocation of this factor by immunofluorescence method directly. Translocation of NF-κB was reduced by AS-6 in LPS-induced RAW cells as observed by the confocal microscopy, showing a specific attenuation of NF-κB nuclear translocation (Figure 5C). Overall, these results indicate that AS-6 can suppress nuclear translocation of NF-κB in LPS-activated murine macrophage cells.

### Effect of AS-6 on TLR4, MyD-88, and MAPK Signaling Pathway

**Figure 6:**
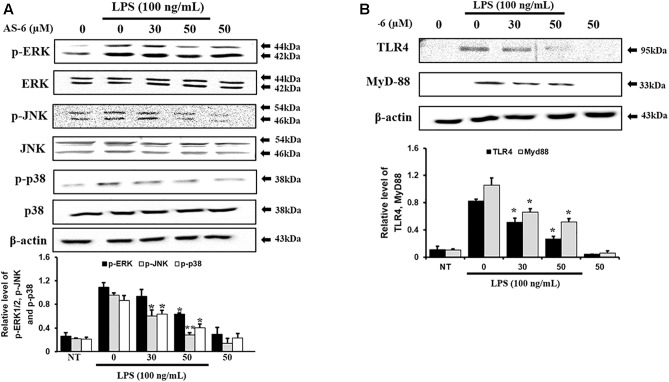
Effect of AS-6 on LPS-activated TLR4, MyD-88, and MAPK signaling pathway in RAW 264.7 cells. RAW cells were pre-treated with AS-6 (50 μM) for 30 min before treatment with 100 ng/mL LPS for 60 min. **(A)** Levels of phosphorylated ERK, p38, and JNK were assessed by Western blot analysis. **(B)** TLR4 and MyD-88 protein levels were determined by Western blot analysis. Levels of ERK, JNK, p38, TLR4, and MyD-88 were indicated by loaded protein as individual control. Results shown are representatives of three independent experiments. Results are presented as mean ± SEM. ^∗^*p* < 0.05 and ^∗∗^*p* < 0.01 indicate significant differences from LPS treated cells. NT, no treatment.

Mitogen-activated protein kinases play important roles in controlling response of inflammation through production of inflammatory mediators (Jiang and Gong, 2000). To assess whether AS-6 affected MAPK phosphorylation in LPS-treated murine macrophages, cells were pre-treated with AS-6 after LPS stimulation and then levels of p-p38, p-ERK and p-JNK were assessed. As shown in Figure 6A, AS-6 decreased expression levels of JNK, ERK and p38 phosphorylation in LPS-induced RAW macrophages. Next, murine macrophages were treated with 0-50 μM AS-6 and then co-treated with LPS (100 ng/mL) for 24 h. TLR4 and MyD-88 protein levels were decreased based on Western blot analysis (Figure 6B). This indicates that AS-6 can inhibit inflammatory response through regulation of TLR4, MyD-88, and MAPKs signaling in LPS-activated murine macrophages.

## Discussion

**Figure 7:**
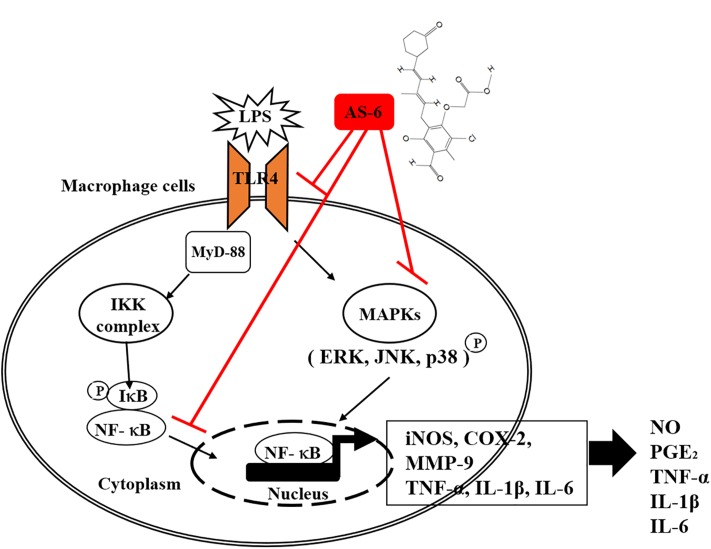
A schematic illustration showing anti-inflammatory responses in RAW 264.7 cells after treatment with AS-6. AS-6 inhibited inflammatory response in RAW 264.7 cells through suppression of TLR4, MyD-88, NF-κB, p-ERK1/2, p-JNK, p-p38, iNOS, COX-2, MMP-9, TNF-a, IL-1B, and IL-6.

There are various chronic inflammatory diseases, including heart disease, cancer, neurological disorders, obesity, and diabetes. Arteriosclerosis, a chronic inflammatory disease, is closely related to the overexpression of pro-inflammatory cytokines and MMP-9 of macrophages (Khatami, 2009). In this study, LPS-stimulated murine macrophages were used as an inflammatory model. Our results indicated that various concentrations of AS-6 decreased MMP-9 expression in LPS-activated murine macrophages. We also performed the effect of AS-6 on LPS-treated inflammatory reactions and explored the potential molecular mechanism. Production of NO and PGE_2_ as major mediators of inflammatory response was inhibited by AS-6 (Figure 2). AS-6 also dose-dependently inhibited the increase of MMP-9, iNOS and COX-2 protein and mRNA levels in LPS-treated RAW 264.7 cells. Furthermore, AS-6 suppressed MMP-9 enzyme activity (Figure 3). During an inflammatory response, mural macrophages are known to secrete inflammatory cytokines such as IL-1β, TNF-α, and IL-6 (Kyriakis and Avruch, 1996). AS-6 attenuated the secretion and expression of pro-inflammatory cytokines in murine macrophages (Figure 4). We then performed experiments to see whether AS-6 could suppress the expression of NF-κB associated with inflammatory responses. AS-6 inhibited LPS-stimulated nuclear translocation of NF-κB in murine macrophages time-dependently (Figure 5). Next, we examined whether AS-6 affected MAPKs phosphorylation. AS-6 decreased expression levels of p-ERK, p-JNK, and p-p38 in LPS-induced murine macrophages. Furthermore, TLR4 and MyD-88 protein levels were assessed by Western blotting (Figure 6). Results showed that AS-6 specifically inhibited the inflammatory response through regulation of TLR4, MyD-88, and MAPKs signaling (Figure 7).

Our aim was to understand the fundamental aspect of AS-6 in action mechanism through basic and applied researches. The present study verified and quantified the suppressive effect of ASC in macrophages. Mechanisms of ASC and other ASC derivatives responsible for their anti-inflammation effects are indefinite objectives at molecular level (Nawata et al., 1969). AS-6, AF, and ASC exert their effects in different ways, most probably because their structural differences allow for specific recognition and inhibition of their target MAPKs. If the structure-functional relationship of compounds is elucidated, such mechanistic clues might be obtained. We found that TLR4 played a role in the down-regulation of MMP-9 upon treatment with AS-6. Data of this study showed that AS-6 inhibited LPS-stimulated inflammatory action by suppressing TLR4/MAPK/NF-κB pathway. In conclusion, AS-6 is a promising inhibitor of chronic inflammation of diseases such as arteriosclerosis and plaque instability involving TLR4. Although the present study has been conducted using *in vitro* experiments with the immune murine macrophage raw cells, *in vivo* experiments with animal model injected with LPS will be required for therapeutic application. Such results will be published in near future elsewhere.

## Data Availability

Publicly available datasets were analyzed in this study. This data can be found here: https://figshare.com/s/adda8ee3d2d007cfa188.

## Author Contributions

C-HK conceived and supervised the study, and administered the project. JP curated the data, acquired funding, developed the software, visualized the data, and wrote the original draft of the manuscript. JP and C-HK performed the formal analysis and validation. JP, FA, EC, and JK investigated the results. JP, S-HH, and HL developed the methodology. JM and Y-CC provided the resources. C-HK, Y-CL, K-TH, and T-WC wrote, reviewed, and edited the manuscript.

## Conflict of Interest Statement

The authors declare that the research was conducted in the absence of any commercial or financial relationships that could be construed as a potential conflict of interest.

## Abbreviations

AS-6: 4-*O*-carboxymethylascochlorin
COX-2: cyclooxygenase-2
DMEM: Dulbecco’s Modified Eagle Medium
DMSO: dimethylsulfoxide
ECL: enhanced chemiluminescence
FBS: fetal bovine serum
FITC: fluorescein isothiocyanate
IL-1β: interleukin-1β
iNOS: inducible nitric oxide synthase
LPS: lipopolysaccharide
MAPKs: mitogen-activated protein kinases
MMP-9: Matrix metallopeptidase 9
MTT: 3-(4,5-dimethylthiazol-2-yl)-2,5-diphenyl tetrazolium bromide
NF-κB: nuclear factor kappa-light-chain-enhancer of activated B cells
PGE_2_: prostaglandin E_2_
TBE: Tris-borate-EDTA
TLR4: Toll-like receptor 4
TNF-α: tumor necrosis factor-α.
